# Quantitative performance of humanized serum and epithelial lining fluid exposures of tigecycline and levofloxacin against a challenge set of *Klebsiella pneumoniae* and *Pseudomonas aeruginosa* in a standardized neutropenic murine pneumonia model

**DOI:** 10.1093/jac/dkae333

**Published:** 2024-10-18

**Authors:** Andrew J Fratoni, Alissa M Padgett, Erin M Duffy, David P Nicolau

**Affiliations:** Center for Anti-Infective Research and Development, Hartford Hospital, 80 Seymour Street, Hartford, CT 06102, USA; Center for Anti-Infective Research and Development, Hartford Hospital, 80 Seymour Street, Hartford, CT 06102, USA; CARB-X, Boston, MA, USA; Center for Anti-Infective Research and Development, Hartford Hospital, 80 Seymour Street, Hartford, CT 06102, USA

## Abstract

**Background:**

Lack of uniformity in infection models complicates preclinical development. The COMBINE protocol has standardized the murine neutropenic pneumonia model. Herein we provide benchmark efficacy data of humanized exposures of tigecycline and levofloxacin in plasma and epithelial lining fluid (ELF) against a collection of *Klebsiella pneumoniae* and *Pseudomonas aeruginosa*.

**Methods:**

Following the COMBINE protocol, plasma and ELF human-simulated regimens (HSRs) of tigecycline 100 mg followed by 50 mg q12h and levofloxacin 750 mg once daily were developed and confirmed in the murine neutropenic pneumonia model. Tigecycline HSRs were tested against seven *K. pneumoniae* isolates. Levofloxacin HSRs were assessed against 10 *K. pneumoniae* and 9 *P. aeruginosa*. The change in cfu/lung over 24 h for each treatment was calculated. Each isolate was tested in duplicate against both the plasma and ELF HSRs on separate experiment days.

**Results:**

Tigecycline 1.8 and 3 mg/kg q12h achieved humanized exposures of serum and ELF, respectively. Levofloxacin 120 and 90 mg/kg q8h led to *f*AUC exposures in plasma and ELF similar to in humans. Both tigecycline regimens were ineffective across the MIC range. Levofloxacin regimens achieved multilog kill against susceptible isolates, and no appreciable cfu/lung reductions in isolates with an MIC of ≥32 mg/L. Differences in cfu/lung were evident between the levofloxacin plasma and ELF HSRs against isolates with MICs of 4 and 8 mg/L.

**Conclusions:**

Administering HSRs of tigecycline and levofloxacin based on both serum/plasma and ELF in the COMBINE pneumonia model resulted in cfu/lung values reasonably aligned with MIC. These data serve as translational benchmarks for future investigations with novel compounds.

## Background

Preclinical pharmacokinetic/pharmacodynamics (PK/PD) models remain a cornerstone of antibiotic development, enabling proof-of-concept efficacy studies and providing valuable insight for the projection of clinical dose selection.^1^ Unfortunately, extensive interlaboratory discrepancies in the methodology of commonly utilized preclinical models predominate the landscape, particularly with pneumonia models.^2^ The collaboration for prevention and treatment of MDR bacterial infections (COMBINE) consortium have proposed a standardized global protocol for the preclinical murine neutropenic pneumonia model in hopes of aligning fundamental elements across laboratories.^3^ Novel therapeutics can benefit from direct comparison with the established standard of care at the preclinical stage of drug development. Establishing benchmark data against a collection of phenotypically and genotypically diverse isolates when treated with humanized exposures of standard-of-care antibiotics across multiple classes in the standardized model can assist in making these comparisons and ensuring model stability and reproducibility across time and location. Importantly for a pneumonia indication, humanized exposures at the target site of infection, pulmonary epithelial lining fluid (ELF), as opposed to plasma, provide an even more translational benchmark. Many investigations in the murine pneumonia model still utilize plasma targets, mostly due to the simplicity of study design, despite potentially unreliable translational application of these targets in the context of agents with discordant ELF penetration between humans and mice. As such, the magnitudes of plasma PK/PD indices required for efficacy determined in a murine pneumonia model may fail to predict the clinical outcome.^4^

Previous efforts have established an isolate collection of *Klebsiella pneumoniae* and *Pseudomonas aeruginosa* suitable for this purpose. These isolates have been phenotypically characterized using broth microdilution to determine modal MIC values, and their viability and the growth stability of untreated controls in the standardized murine neutropenic pneumonia model have been reported.^5^ This manuscript details the development of human-simulated regimens (HSRs) of tigecycline and levofloxacin in both plasma and pulmonary ELF in the model and describes the change in cfu/lung with these regimens against Gram-negative isolates.

## Materials and methods

### Antimicrobial agents

Commercial vials of tigecycline were reconstituted for *in vivo* studies and diluted further in 0.9% saline to deliver weight-based dosing to the mice. For levofloxacin, analytical grade powder (Sigma–Aldrich) was acquired and dissolved in sterile water with the addition of 0.1 M NaOH.

### Bacterial isolates

*K. pneumoniae* and *P. aeruginosa* isolates were sourced from the isolate repository at the Center for Anti-Infective Research and Development (CAIRD) (Hartford, CT, USA), the CDC and FDA Antibiotic Resistance Isolate Bank (CDC Bank) (Atlanta, GA, USA), the Paul Ehrlich Institute (PEI) (Berlin, Germany), and the Leibniz Institute (DSMZ) (Brunswick, Germany). The phenotypic and known genotypic profiles of these isolates were previously determined and are provided in Table 1.^5^ Prior to experimentation, each isolate was subcultured twice on Trypticase soy agar with 5% sheep blood (Becton Dickinson and Co., Sparks, MD, USA) and incubated at 37°C for approximately 16 h. Bacterial colonies from the overnight culture plate were suspended in normal saline to a McFarland target of ∼2.5 and further diluted in saline as necessary to produce the final inoculum.

**Table 1.** Phenotypic and known genotypic profiles of *K. pneumoniae* and *P. aeruginosa* isolates

			MIC (mg/L)		Known genotypic information
	Isolate origin	Isolate ID	TGC	LVX	
*K. pneumoniae*	CDC Bank	523	1	4	aadA1, aph(3′)-Ia, dfrA1, KPC-2, sul1
	CDC Bank	542	4	>32	aac(6′)-Ib, aadA2, catA1, dfrA12, EMRD, KDEA, mph(A), oqxA, oqxB, SHV-12, sul1
	CDC Bank	558	0.5	8	aac(3)-IId, aac(6′)-Ib-cr, aadA1, aadA2, armA, ARR-2, catA1, CTX-M-15, dfrA12, dfrA14, EMRD, ere(A), fosA5, KDEA, OXA-181, SHV-26, sul1, sul2, tet(A), tet(R)
	CDC Bank	560	1	>32	aac(3)-IId, aac(6′)-Ib-AKT, aadA1, armA, ARR-2, ble-MBL, catA1, catB4, cmlA5, CMY-4, CTX-M-15, dfrA1, EMRD, ere(A), fosA, KDEA, mph(E), msr(E), NDM-1, oqxA, oqxB20, OXA-1, OXA-9, sat2, SHV-100, sul1, TEM-1A
	CDC Bank	831	8	4	aac(3)-IIa, aph(3′)-Ib, aph(6)-Id, catB4, CTX-M-15, dfrA14, OXA-1, QnrB1, SHV-187, sul2, TEM-1B, tet(A), tet(R)
	CDC Bank	848	4	>32	aac(3)-IIa, aac(6′)-Ib-cr, catB4, CTX-M-15, dfrA1, Omp35, OmpK35, oqxA, oqxB, OXA-1, OXA-48, QnrS1, SHV-11, sul1, TEM-1B, tet(A), tet(R)
	PEI	Kp C1.112	0.25	≤0.063	Unknown
	PEI	Kp C1.113	0.25	≤0.063	Unknown
	PEI	Kp C1.147	1	32	CTX-M, OXA-48-like
	PEI	Kp C1.151	1	>32	KPC
	DSMZ	30104	0.25	0.063	Unknown
*P. aeruginosa*	CDC Bank	354	N/A	8	aadA11, ant(2′)-la, aph(6)-Id, dfrB1, strA
	CDC Bank	459	N/A	8	OXA-50
	CDC Bank	516	N/A	0.5	KPC-2
	CDC Bank	767	N/A	32	GES-20
	PEI	Pa 88198	N/A	4	Unknown
	PEI	Pa 88276	N/A	1	Unknown
	PEI	Pa 88342	N/A	1	Unknown
	CAIRD	PSA INT-2-41	N/A	>32	aac(6′)-Ib, aac(6′)-Ib-cr, aadA6, aph(3′)-IIb, bla_CTX-M-2_, bla_OXA-488_, bla_PDC-35_, catB7, fosA, qacE, sul1
	CAIRD	PSA INT-4-99	N/A	32	aac(6′)-Ib-cr, aadA6, aph(3′)-IIb, bla_OXA-14_, bla_OXA-488_, bla_PDC-35_, catB7, cml, cmlA1, fosA, qacE,sul1

LVX, levofloxacin; MEM, meropenem; FDC, cefiderocol; TOB, tobramycin; N/A, not applicable.

### In vivo murine neutropenic pneumonia model

The details of the COMBINE protocol and model specificities have been previously published.^3^ In brief, specific-pathogen-free CD-1, female mice 6–8 weeks old were acquired from Charles River Laboratories, Inc. (Raleigh, NC, USA). All animals were allowed to acclimatize for 72 h prior to any study procedures and were housed as groups of six at controlled room temperature in HEPA-filtered cages (Innovive, San Diego, CA, USA). Study rooms were maintained with diurnal cycles (12 h light/12 h dark) and food and water were provided *ad libitum*. Prior to inoculation, neutropenia was induced by administering 150 mg/kg of intraperitoneal (IP) cyclophosphamide (0.2 mL) on Day −4 and 100 mg/kg on Day −1. In addition, a predictable degree of renal impairment was produced using 5 mg/kg uranyl nitrate administered IP (0.2 mL) on Day −3. Mice were anaesthetized using inhaled isoflurane, manually restrained upright, and infected with 50 µL of bacterial suspension via the nares. Inoculums were prepared to ∼10^8^ and 10^7^ cfu/mL for *K. pneumoniae* and *P. aeruginosa*, respectively, to deliver 10^7^ and 10^6^ cfu/lung, respectively, which are necessary for model performance as previously described.^5^ Dosing was initiated 2 h after inoculation.

### PK studies

#### Ex vivo tigecycline serum protein-binding studies

All PK studies undertaken to develop the HSRs (including protein-binding studies) were performed in mice infected with *K. pneumoniae* (CDC 851), and mice were handled as described above. Escalating single doses of tigecycline (2.25, 4.5 and 9 mg/kg) were administered subcutaneously to determine tigecycline serum protein binding. Triplicate pooled serum and ultrafiltrate samples were collected at 1 h (5 mice per replicate, 15 mice per dose) and stored at −80°C until concentration determination. Whole blood was allowed to clot and then subsequently centrifuged at 4°C at 3000 × **g** for 10 min. Serum was separated (total serum) and 900 µL was added to an ultrafiltration device (Centrifree^®^, Merck Millipore Ltd., Ireland) and centrifuged using a fixed rotor at 10°C at 2000 × **g** for an additional 45 min to obtain protein-free ultrafiltrate. The triplicate free and total tigecycline concentrations were averaged and then free fractions were calculated using: free fraction = Concentration_ultrafiltrate_/Concentration_total serum_.

#### Human-simulated exposure PK studies

PK studies were undertaken to establish HSRs that approximate *f*AUC_0–24_ exposures achieved after IV administration of tigecycline 100 mg loading dose followed by 50 mg q12h and levofloxacin 750 mg q24h in humans. Separate HSRs were developed for serum (tigecycline), plasma (levofloxacin) and pulmonary ELF (both). The blood matrices for tigecycline and levofloxacin of serum and plasma, respectively, were selected to align with human PK studies.^6,7^ At predefined timepoints, groups of six mice had blood sampled via retro-orbital bleeding (two timepoints, under anaesthesia with isoflurane 2%–3% v/v in 100% oxygen via inhalation) and cardiac puncture (one terminal timepoint). Proparacaine hydrochloride ophthalmic solution 0.5% was applied to the eye (1–2 drops) after blood sampling via retro-orbital bleeding. The volume of blood collected was 0.15 mL per sample via retro-orbital bleeding with subsequent fluid replacement using 0.2 mL normal saline given IP. Unlike protein-binding studies, samples were not pooled and the exposure in each individual mouse was assessed. Tigecycline free serum concentrations were determined from the total utilizing the protein-binding percentage determined presently. Levofloxacin free plasma exposures were estimated from the total by assuming 30% protein binding, which has shown to be consistent across humans and multiple animal species, including mice.^8,9^ Following blood collection by cardiac puncture, but prior to cervical dislocation, bronchoalveolar lavage (BAL) fluid was collected from the mice at the same timepoints. A catheter was inserted into the trachea of the mice, and lungs were lavaged with four aliquots of 0.4 mL of normal saline. For ELF concentration determination, urea correction was applied to BAL fluid concentrations. Due to the lack of albumin in BAL fluid, total ELF concentrations were considered free (unbound). *f*AUC for each dose was determined using the trapezoid method and multiplied by the number of doses throughout the 24 h experiment to determine *f*AUC_0–24_. Previously reported tigecycline and levofloxacin serum/plasma HSR regimens developed in alternative murine models were used as a baseline.^10,11^ Mathematical modifications were made to the baseline regimens as necessary to achieve translational *f*AUC exposures and repeat confirmatory PK studies were undertaken for both serum/plasma and ELF. While HSRs were developed and confirmed prior to *in vivo* efficacy studies, additional PK studies were undertaken on the same day as *in vivo* efficacy studies using the same methods and predefined timepoints to assess interday and inter-isolate variability.

The sample size calculation was performed using nQuery Advisor based on the following: (i) the mean % coefficient of variance (CV) of the PK parameter for typical antibiotics is usually less than 30%; and (ii) a two-sided 95% CI with 80% probability will have an interval that extends no more than 1 SD from the observed mean. As a result, the sample size of six mice per timepoint is sufficient for the assessment of drug disposition.

### In vivo efficacy studies

Controls were sacrificed just prior to antibiotic initiation (0 h controls) and 24 h later. All groups contained six mice except for 24 h controls, which contained 4–6 mice. Plasma (or serum) and pulmonary ELF HSRs were administered over 24 h (or equal volume of normal saline in controls), then animals were euthanized by CO_2_ asphyxiation followed by cervical dislocation. Lungs from individual animals were aseptically harvested and homogenized in normal saline. Homogenized tissue was then serially diluted and 50 µL was plated for cfu/lung quantification (lower limit of quantification of 1 × 10^2^ cfu/lung). The change in cfu/lung over 24 h for each treatment was calculated relative to the initial bacterial burden. Each isolate was tested in duplicate against both the plasma and ELF HSRs on separate experiment days to incorporate interday model variability. The raw data from individual experiments were combined and analysed together. Tigecycline serum and ELF HSRs were tested against seven *K. pneumoniae* isolates with tigecycline MICs previously determined by broth microdilution ranging from 0.25 to 8 mg/L. Levofloxacin plasma and ELF HSRs were assessed against 10 *K. pneumoniae* isolates (levofloxacin MIC range: ≤ 0.063 to >32 mg/L) and 9 *P. aeruginosa* isolates (levofloxacin MIC range: 0.5 to >32 mg/L).

Sample size was calculated as follows: (i) for typical antimicrobial agents, optimal dosing regimens usually produce approximately 2–3 log decrease in bacterial density with 40% CV; and (ii) in order to have an observed mean that deviates from the true mean by no more than 1 SD using a two-sided 95% CI with 80% probability, *n* = 6 datapoints are required.

### Analytical procedures

All compounds were analysed using a Waters Acquity UPLC H-Class system with tandem TQ-XS mass spectrometer (LC-MS/MS) equipped with an Acquity UPLC BEH C18, 1.7 μm, 2.1 × 50 mm column. All reagents were obtained from commercial sources and used without further purification.

Concentrations of levofloxacin in BAL and murine K_2_EDTA plasma were determined with validated UPLC methods using levofloxacin-*d*_8_ as the internal standard. The concentration range for both levofloxacin methods was 0.01–100 μg/mL. Mean interday CV for low, medium and high values of levofloxacin in saline were 10.4%, 7.2% and 12.3%, respectively. Mean interday CV for low, medium and high values of levofloxacin in murine K_2_EDTA plasma were 6.3%, 5.2% and 7.6%, respectively.

Concentrations of tigecycline in BAL and murine serum were determined with validated UPLC methods using tigecycline-*d*_9_ as the internal standard. The concentration range for the tigecycline in the BAL method was 1–2500 ng/mL, while the concentration range for the tigecycline in serum method was 0.05–25 μg/mL. Mean interday CV for low, medium and high values of tigecycline in saline were 8.5%, 4.2% and 7.5%, respectively. Mean interday CV for low, medium and high values of tigecycline in murine serum were 9.9%, 11.7% and 9.8%, respectively.

Urea concentrations in BAL and murine K_2_EDTA plasma and serum were determined with a validated UPLC method using standards in saline (5–500 μg/mL) and [^13^C]-urea as the internal standard. The internal standard was diluted with 3:4 acetonitrile/water to yield a 950 ng/mL solution of [^13^C]-urea for protein precipitation. Compounds were monitored using an ESI probe in positive acquisition mode. The quantitative mass transition for urea was 61.0 → 43.8. The quantitative mass transition for [^13^C]-urea was 62.0 → 45.0. For the preparation of all standards and samples, 630 μL of protein precipitation solution was added to a well plate containing 30 μL of standard or sample. Nominal concentrations of the low, medium and high quality controls for urea in saline were 7.5, 75 and 400 μg/mL, respectively. Mean interday CV for low, medium and high values of urea were 9.6%, 9.1% and 9.4%, respectively.

### Ethics and laboratory animals

Animals were maintained and utilized in accordance with National Research Council recommendations. The study protocol was reviewed and approved by the Institutional Animal Care and Use Committee at Hartford Hospital (Assurance #A3185-01).

## Results

### PK studies

#### Ex vivo tigecycline serum protein-binding studies

Similar to previous findings, the percentage of tigecycline bound to protein was dose-dependent.^12^ Mean percentages of protein binding (±SD) were 15.9% (5.0%), 50.5% (18.5%) and 41.7% (15.1%) at the 1 h timepoint after receiving doses of 2.25, 4.5 and 9 mg/kg, respectively. For the tigecycline serum HSR, all total concentrations in the profile were corrected for binding using 15.9% as the exposures were closest to the 2.25 mg/kg dose.

#### Human-simulated exposure PK studies

In the COMBINE murine neutropenic pneumonia model, administration of tigecycline 1.8 mg/kg every 12 h delivered in 0.1 mL subcutaneous injections achieved an *f*AUC_0–24_ of 2.38 mg·h/L after correcting for 15.9% protein binding. This free drug exposure is consistent with findings in patients receiving 100 mg followed by 50 mg every 12 h with ventilator-associated pneumonia (VAP) and exceeds exposures observed in healthy volunteers (Table 2).^13,14^ The penetration into ELF was observed to be lesser in mice than in man, necessitating a higher dose of 3 mg/kg every 12 h to achieve an ELF AUC_0–24_ of 5.68 mg·h/L, which is comparable to exposures seen in both VAP patients and healthy volunteers.^13,14^ The observed tigecycline concentrations from repeat PK studies performed concurrently with *in vivo* efficacy studies are available in Figure S1 (available as Supplementary data at *JAC* Online).

**Table 2.** Tigecycline *f*AUC_0–24_ exposures in humans and mice receiving HSRs

Tigecycline regimen	Species (matrix)	*f*AUC_0–24_ (mg·h/L) Mean ± SD
100 mg LD, 50 mg q12h maintenance	Human (serum)	0.88 ± 0.17 (HV)^13^
		3.24 ± 3.09 (VAP)^14^
1.8 mg/kg q12h (serum HSR)	Murine (serum)	2.38
100 mg LD, 50 mg q12h maintenance	Human (ELF)	6.32 (HV)^a^
		7.13 ± 2.61 (VAP)
3 mg/kg q12h (ELF HSR)	Murine (ELF)	5.68

LD, loading dose; HV, healthy volunteer; VAP, ventilator-associated pneumonia.

^a^SD not provided in the reference.

**Figure 1.**
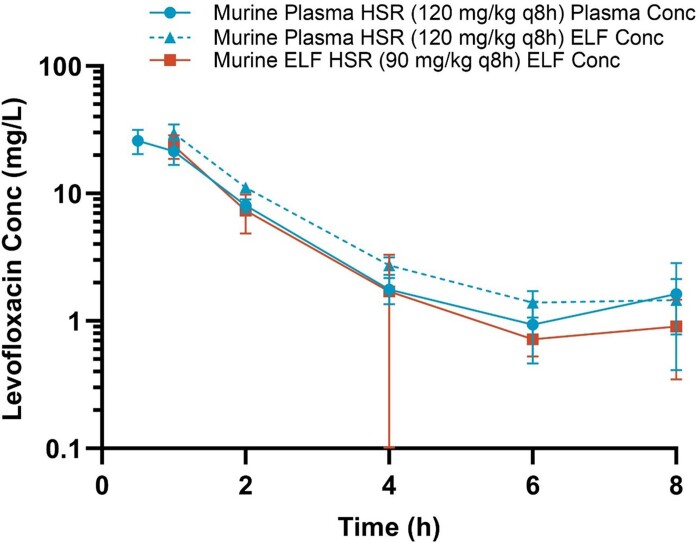
Levofloxacin exposures in the COMBINE murine neutropenic pneumonia model following administration of plasma and ELF HSRs of 750 mg every 24 h. Conc, concentration. This figure appears in colour in the online version of *JAC* and in black and white in the print version of *JAC*.

Administration of 120 mg/kg levofloxacin every 8 h in the model yielded plasma AUC_0–24_ exposure of 145 mg·h/L in mice, which is well matched with the mean plasma exposure (140 mg·h/L) observed in infected patients with acute exacerbation of chronic bronchitis receiving 750 mg orally every 24 h.^6^ Assuming 30% protein binding, the resultant plasma *f*AUC_0–24_ was 102 mg·h/L. Conversely to tigecycline, ELF penetration was greater in infected mice relative to humans with infections. The administration of the levofloxacin plasma HSR (120 mg/kg every 8 h) resulted in ELF AUC_0–24_ exposure of 168 mg·h/L, which is ∼40% higher than the median exposure of 119 mg·h/L observed in the ELF of infected humans.^6^ Therefore, a reduction in dose to 90 mg/kg every 8 h was required to humanize ELF exposures in mice, with a resulting AUC_0–24_ of 129 mg·h/L (Figure 1). Data derived from repeated PK studies of the levofloxacin HSRs undertaken alongside efficacy studies are presented in Figure S2.

### In vivo efficacy studies

#### Tigecycline serum and ELF HSR efficacy

**Figure 2.**
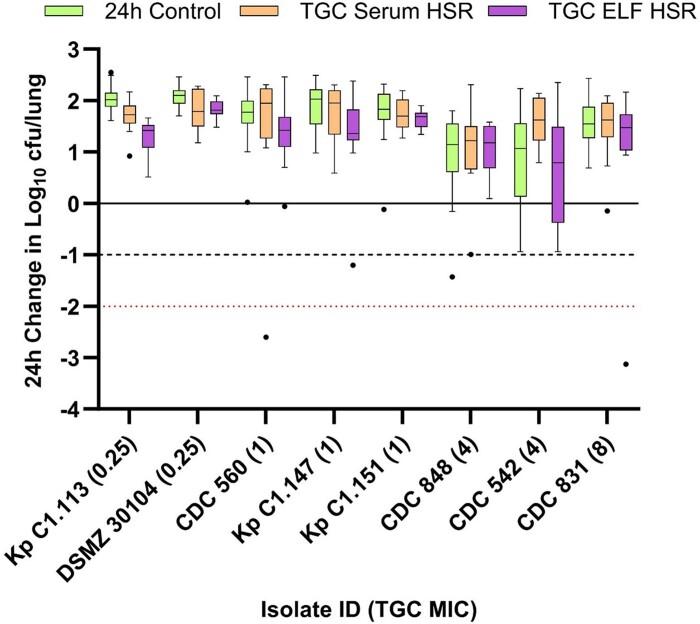
Cfu/lung data following administration of humanized tigecycline (TGC) (100 mg followed by 50 mg every 12 h) serum and ELF exposures in the COMBINE murine neutropenic pneumonia model against *K. pneumoniae* isolates. Boxes represent IQRs, with horizontal lines denoting the medians. Whiskers were determined by Tukey’s test and outliers are displayed as individual dots. The solid line denotes stasis; the dashed line denotes 1 log kill; the red dotted line denotes 2 log kill.

The mean ± SD baseline bacterial burden, and 24 h change in log_10_ cfu/lung in controls relative to baseline across the seven *K. pneumoniae* isolates tested were 7.34 ± 0.09 and 1.65 ± 0.44 log_10_ cfu/lung, respectively. Inoculum cfu/mL, baseline bacterial burden and growth in 24 h controls are available on an individual isolate basis in Table S1. The 24 h change in log_10_ cfu/lung after receiving saline control, tigecycline serum HSR and tigecycline ELF HSR for each of the seven isolates is presented in Figure 2. Among all isolates, net growth was observed on both the serum and ELF HSRs, with minimal differentiation between the HSRs when simulating the currently licensed tigecycline dose of 100 mg followed by 50 mg every 12 h.

#### Levofloxacin plasma and ELF HSR efficacy

##### K. pneumoniae

**Figure 3.**
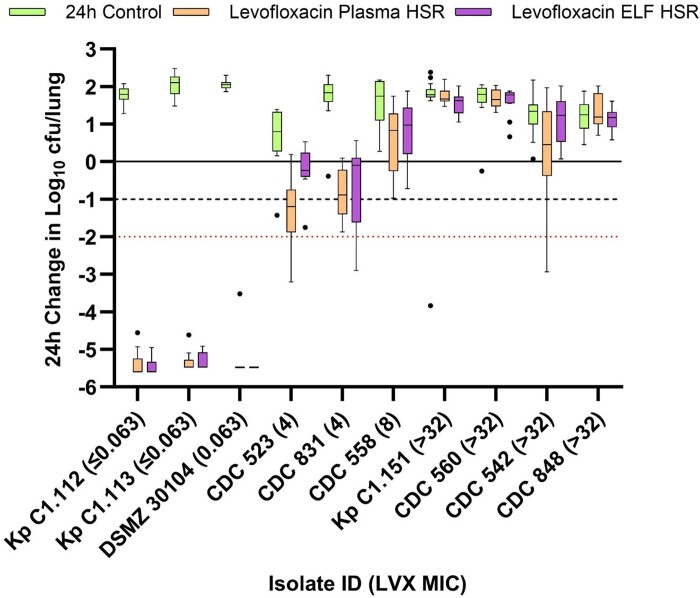
Cfu/lung data following administration of humanized levofloxacin (750 mg every 24 h) plasma and ELF exposures in the COMBINE murine neutropenic pneumonia model against *K. pneumoniae* isolates. Boxes represent IQRs with horizontal lines denoting the medians. Whiskers were determined by Tukey’s test and outliers are displayed as individual dots. The solid line denotes stasis; the dashed line denotes 1 log kill; the red dotted line denotes 2 log kill.

The mean ± SD baseline bacterial burden and 24 h change in log_10_ cfu/lung in controls relative to baseline across the 10 *K. pneumoniae* isolates tested were 7.37 ± 0.22 and 1.62 ± 0.40 log_10_ cfu/lung, respectively. The 24 h change in log_10_ cfu/lung after receiving saline control, levofloxacin plasma HSR and levofloxacin ELF HSR for each of the 10 isolates is presented in Figure 3. The three isolates categorically susceptible to levofloxacin experienced multilog kill when exposed to either HSR. Among the two isolates with an MIC of 4 mg/L, there was clear differentiation in the efficacy of the plasma and ELF HSR regimens. When the levofloxacin exposure at the target site of infection (i.e. ELF) was matched, the 1 log kill efficacy target was not achieved. When matched solely on plasma exposures, both isolates neared or exceeded 1 log kill. The remaining isolates with an MIC of >4 mg/L experienced mean growth relative to the corresponding baseline bacterial burden, regardless of which levofloxacin HSR was administered.

##### P. aeruginosa

**Figure 4.**
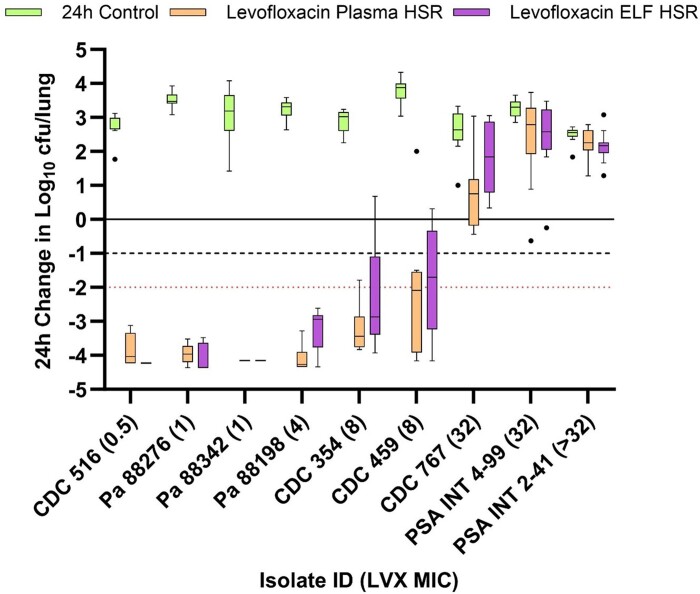
Cfu/lung data following administration of humanized levofloxacin (750 mg every 24 h) plasma and ELF exposures in the COMBINE murine neutropenic pneumonia model against *P. aeruginosa* isolates. Boxes represent IQRs with horizontal lines denoting the medians. Whiskers were determined by Tukey’s test and outliers are displayed as individual dots. The solid line denotes stasis; the dashed line denotes 1 log kill; the red dotted line denotes 2 log kill.

Among the nine *P. aeruginosa* isolates tested in the model against levofloxacin, the mean ± SD baseline bacterial burden and 24 h change in log_10_ cfu/lung in controls relative to baseline were 6.13 ± 0.22 and 3.10 ± 0.38 log_10_ cfu/lung, respectively. Figure 4 displays the 24 h change in log_10_ cfu/lung after receiving saline control, levofloxacin plasma HSR and levofloxacin ELF HSR for each isolate. Multilog killing was seen with the levofloxacin HSR of each matrix in the four isolates with an MIC of ≤4 mg/L (the resistance breakpoint). There was considerable variability in cfu/lung in the two isolates with an MIC of 8 mg/L, with the plasma HSR achieving larger reductions relative to ELF HSR. As expected based on phenotype, the remaining isolates with an MIC of 32 mg/L or greater experienced net growth after administration of either levofloxacin HSR.

## Discussion

Harmonization of crucial elements of *in vivo* infection models is imperative for making reasonable comparisons between compounds and laboratories, especially when assessing against the same cohort of isolates. In accordance with the COMBINE protocol for the murine neutropenic pneumonia model, we developed and confirmed serum (tigecycline), plasma (levofloxacin) and pulmonary HSRs (both). While these HSRs were developed using the methodology and drug products as noted herein, confirmatory PK studies should be undertaken prior to future use of our mg/kg dosing schemes as differences in the drug products (i.e. pharmaceutical versus analytical grade), formulations and manufacturers may result in markedly different drug exposures in each of the biological target sites that have been profiled.

These HSRs were subsequently administered to mice infected with various *K. pneumoniae* and *P. aeruginosa* strains, with every isolate tested in duplicate against HSRs of both matrixes. As anticipated, the tigecycline simulated exposures were ineffective in producing cfu/lung reductions against this collection of *K. pneumoniae* isolates. Levofloxacin displayed greater ELF penetration in this murine model relative to humans, necessitating a lower mg/kg dose to simulate human ELF concentration-versus-time profile in relation to the plasma HSR. Both HSRs for levofloxacin achieved multilog kill against all susceptible isolates. The plasma HSR, which overexposes the ELF, led to greater cfu reductions than the ELF HSR in isolates at the resistance breakpoint or a few doubling dilutions greater, underscoring the importance of matching exposures at the site of infection so as to not overpredict activity or efficacy in cases where the penetration is discordant between matrixes.

Of note, these experiments developed humanized serum and ELF exposures of tigecycline commensurate with the FDA-approved dose of 100 mg followed by 50 mg every 12 h. These exposures have demonstrated a lack of clinical efficacy when used to treat Gram-negative bacterial pneumonia, which is consistent with the known PK/PD properties of the drug.^15–17^ Therefore, we introduced tigecycline at these exposures as a negative control in the model. Notably, many clinicians utilize an off-label high-dose regimen of 200 mg followed by 100 mg every 12 h, which has been shown to improve clinical outcomes relative to labelled dosing in VAP or hospital-acquired pneumonia.^18,19^ This increase in dose better optimizes exposures in regard to PK/PD targets but importantly is not represented in these studies.

As reported previously, when discovered in an earlier phase of model development and validation, it should be noted that the initial bacterial burden between *K. pneumoniae* and *P. aeruginosa* isolates differs.^5^ While not ideal, it is necessary to start *K. pneumoniae* at a higher inoculum (≥7 log_10_ cfu/lung) to better ensure the consistent ability for control animals to achieve ≥1 log of growth, and extending *P. aeruginosa* initial bacterial burden beyond 6 log_10_ cfu/lung is liable to lead to overt infection-related mortality before 8 h, where drug therapy is unlikely to have any meaningful benefits. This ∼1 log difference in initial bacterial burden between bacterial species could theoretically lead to differences in PD profiling in terms of magnitude of exposure needed, particularly against antibiotic classes that are prone to an inoculum effect, albeit inoculum effects are generally described when assessing multilog differences *in vitro* as opposed to 1 log in this *in vivo* murine pneumonia model.

As the purpose of these experiments was to set quantitative cfu/lung benchmarks in the COMBINE model using the selected isolates that could be replicated by other laboratories, it was imperative that we could replicate our own findings and understand interday variability on an intralaboratory level. Therefore, a strength of this study was the assessment of each HSR against each isolate on two separate study days. These data from separate days were combined and analysed together as one to incorporate the variability between separate investigations and provide a more reasonable benchmark for other investigators to match. This also served as an indirect quality control between each study day, as discordant results between the original and repeated investigation of each isolate and regimen would signal potential error in study methodology. Overtly discordant results were not observed for the efficacy studies of either drug. Interday study variability was generally within 0.5 log_10_ cfu/lung in either direction, which is a reasonable target for external investigators looking to replicate these findings as the data presented are inclusive of our study-to-study variability.

Similarly, additional efforts were taken to quantify drug exposures during the *in vivo* efficacy assessments after separate PK validation of the HSRs. Relative to original confirmatory PK data, the concentrations obtained in the ELF for tigecycline during the efficacy assessments were very reproducible, albeit serum data were more variable. For levofloxacin, the concentration-versus-time profiles with CDC 831 (*K. pneumoniae*) suggest similar volume of distribution and clearance compared with original PK confirmatory studies, which were also performed in animals infected with a *K. pneumoniae* isolate. However, infection with CDC 767 (*P. aeruginosa*) appeared to lead to a decrease in drug clearance, but again a similar volume of distribution. This difference in clearance may be linked to the mortality observed in animals infected with CDC 767 relative to the two *K. pneumoniae* isolates. As animals become increasingly septic, it would be expected that their renal function declines, leading to decreased drug clearance of renally excreted compounds like levofloxacin. Importantly, drug concentrations over the initial 8–12 h of the model before overt sepsis determine the majority of activity (or lack thereof), so reasonable differences in the exposure of the later portion of the infection model are expected variability.

In this model, both antibiotics tested had ELF penetration discordant from humans. Interestingly, these discordances were in opposing directions, with tigecycline ELF underexposed and levofloxacin ELF overexposed when serum/plasma human profiles were simulated. While these differences between species may seem trivial, the implications have been proven to be serious when unaccounted for, as demonstrated by ceftobiprole.^4,20^ Simply stated, using plasma exposure targets from a murine pneumonia model cannot reliably predict the efficacy or microbiological effect in humans. To enhance clinical translation, murine pneumonia models must quantify ELF exposure. Undoubtedly, PK sampling and analysis of the ELF is more complicated, costly and time-consuming. It also introduces additional variables such as urea concentrations in both plasma and ELF for dilution correction. However, the importance of its characterization cannot be overstated. Another point to consider for clinical translation is the source of human ELF data. Similar to how we conduct ELF PK in infected mice, ideally human PK ought to be generated in patients with infection also. While ELF exposure of levofloxacin was thought to be multitudes higher than in plasma based on healthy volunteer data, it was later demonstrated that the median penetration in infected patients was 0.9 as opposed to 2.4 in healthy volunteers.^6^ Developing an ELF HSR based on healthy volunteer data for levofloxacin would have resulted in marked exposure differences and lessened the clinical translation of the model.

The protein-binding characteristics of the tetracyclines are complex and the literature is mixed.^21–23^ The class is generally considered concentration-dependent but unlike conventional drug wisdom, does not display inverse proportionality where lower concentrations are bound at a higher percentage and higher concentrations are bound at a lower percentage. Instead, protein binding for this drug class increases with increasing exposure.^24,25^ We found similar findings in this model. For protein-binding assessments, *ex vivo* methods are ideal as they maintain a higher level of biological complexity relative to *in vitro* experiments. While murine serum could have been purchased and spiked with tigecycline to determine *in vitro* protein binding, our approach of preparing, infecting and dosing mice consistent with our efficacy studies and sampling directly from the model is likely a more accurate representation of protein binding within the model. This *ex vivo* methodology eliminates variables that need to be accounted for when running *in vitro* assessments such as duration of drug exposure with the matrix, temperature and any additives or pretreatment to the serum.

In summary, this study developed serum (tigecycline), plasma (levofloxacin) and ELF HSRs (both) in the COMBINE murine neutropenic pneumonia model. These regimens were tested in duplicate against a previously characterized challenge set of *K. pneumoniae* and *P. aeruginosa* strains, with efficacy (at least for the ELF HSRs) that could be reasonably anticipated based on MIC. Meropenem, cefiderocol and tobramycin are three additional antibiotics that will undergo similar plasma and ELF HSR development and quantitative cfu/lung assessment in the COMBINE model against the same collection of isolates described previously to provide robust benchmarks using compounds representative of many antibiotic classes for future compound development.

## Supplementary Material

dkae333_Supplementary_Data

## Supplementary data

Figures S1 and S2 and Table S1 are available as Supplementary data at *JAC* Online.
