# Supplementary material for: Quantitative performance of humanized serum and epithelial lining fluid exposures of tigecycline and levofloxacin against a challenge set of Klebsiella pneumoniae and Pseudomonas aeruginosa in a standardized neutropenic murine pneumonia model
Source: J Antimicrob Chemother. 2024 Oct 18;79(12):3142–9. doi: 10.1093/jac/dkae333 (PMC11638853; doi:10.1093/jac/dkae333)

**Quantitative Performance of Humanized Serum and Epithelial Lining Fluid Exposures of Tigecycline and Levofloxacin Against a Challenge Set of *Klebsiella pneumoniae* and *Pseudomonas aeruginosa* in a Standardized Neutropenic Murine Pneumonia Model**

*Journal of Antimicrobial Chemotherapy*

Andrew J. FRATONI, Alissa M. PADGETT, Erin M. DUFFY, David P. NICOLAU

Supplemental Data File

Version 8.21.2024

**Table S1.** Inoculum CFU/mL, initial bacterial burden, and growth at 24h in controls for all isolates tested in the COMBINE neutropenic pneumonia model against tigecycline and levofloxacin human simulated regimens.

|  | **Isolate Origin** | **Isolate ID** | **Inoculum CFU/mL (mean±SD)** | **Initial Bacterial Burden CFU/Lung (mean±SD)** | **Control Growth at 24h CFU/Lung (mean±SD)** |
| --- | --- | --- | --- | --- | --- |
| *Klebsiella pneumoniae* | CDC Bank | 523 | 8.28**±**0.21 | 7.16±0.54 | 8.38±0.92 |
|  | CDC Bank | 542 | 8.30±0.18 | 7.29±0.22 | 8.32±0.81 |
|  | CDC Bank | 558 | 8.20±0.14 | 6.84±0.29 | 8.66±0.62 |
|  | CDC Bank | 560 | 8.30±0.20 | 7.38±0.30 | 9.06±0.52 |
|  | CDC Bank | 831 | 8.38±0.20 | 7.25±0.34 | 8.93±0.51 |
|  | CDC Bank | 848 | 8.46±0.28 | 7.39±0.28 | 8.47±0.62 |
|  | PEI | Kp C1.112 | 8.51±0.30 | 7.60±0.32 | 9.38±0.22 |
|  | PEI | Kp C1.113 | 8.39±0.28 | 7.38±0.64 | 9.53±0.26 |
|  | PEI | Kp C1.147 | 8.36±0.32 | 7.27±0.26 | 9.18±0.44 |
|  | PEI | Kp C1.151 | 8.39±0.18 | 7.49±0.22 | 9.15±0.96 |
|  | DSMZ | 30104 | 8.26±0.27 | 7.40±0.31 | 9.48±0.16 |
| *Pseudomonas aeruginosa* | CDC Bank | 354 | 7.39±0.16 | 6.13±0.34 | 9.03±0.32 |
|  | CDC Bank | 459 | 7.59±0.24 | 6.16±0.15 | 9.92±0.36 |
|  | CDC Bank | 516 | 7.41±0.03 | 6.23±0.16 | 9.13±0.17 |
|  | CDC Bank | 767 | 7.42±0.04 | 5.99±0.17 | 8.74±0.39 |
|  | PEI | Pa 88198 | 7.74±0.22 | 6.34±0.12 | 9.57±0.28 |
|  | PEI | Pa 88276 | 7.75±0.21 | 6.36±0.15 | 9.89±0.22 |
|  | PEI | Pa 88342 | 7.78±0.61 | 6.15±0.17 | 9.19±0.79 |
|  | CAIRD | PSA INT-2-41 | 7.21±0.20 | 6.16±0.12 | 8.70±0.11 |
|  | CAIRD | PSA INT-4-99 | 7.45±0.06 | 5.62±0.36 | 8.89±0.25 |

**Figure S1.** (A) Tigecycline free serum concentration versus time profiles after administration of 1.8 mg/kg every 12h and (B) tigecycline pulmonary epithelial lining fluid concentration versus time profiles after administration of 3 mg/kg every 12h during original human simulated regimen confirmatory pharmacokinetic study and spot-checked concurrent with *in vivo* efficacy studies.


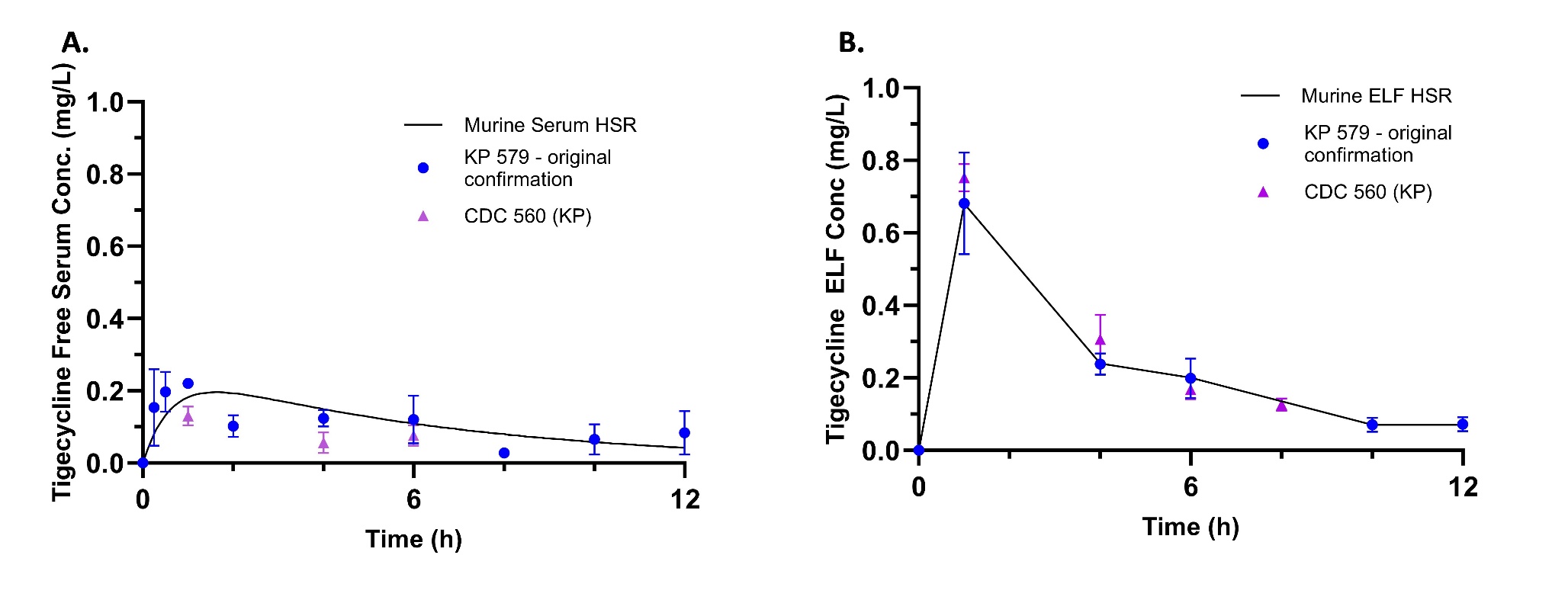


**Figure S2.** (A) Levofloxacin plasma concentration versus time profiles after administration of 120 mg/kg every 8h and (B) levofloxacin pulmonary epithelial lining fluid concentration versus time profiles after administration of 90 mg/kg every 8h during original human simulated regimen confirmatory pharmacokinetic study and spot-checked concurrent with *in vivo* efficacy studies.


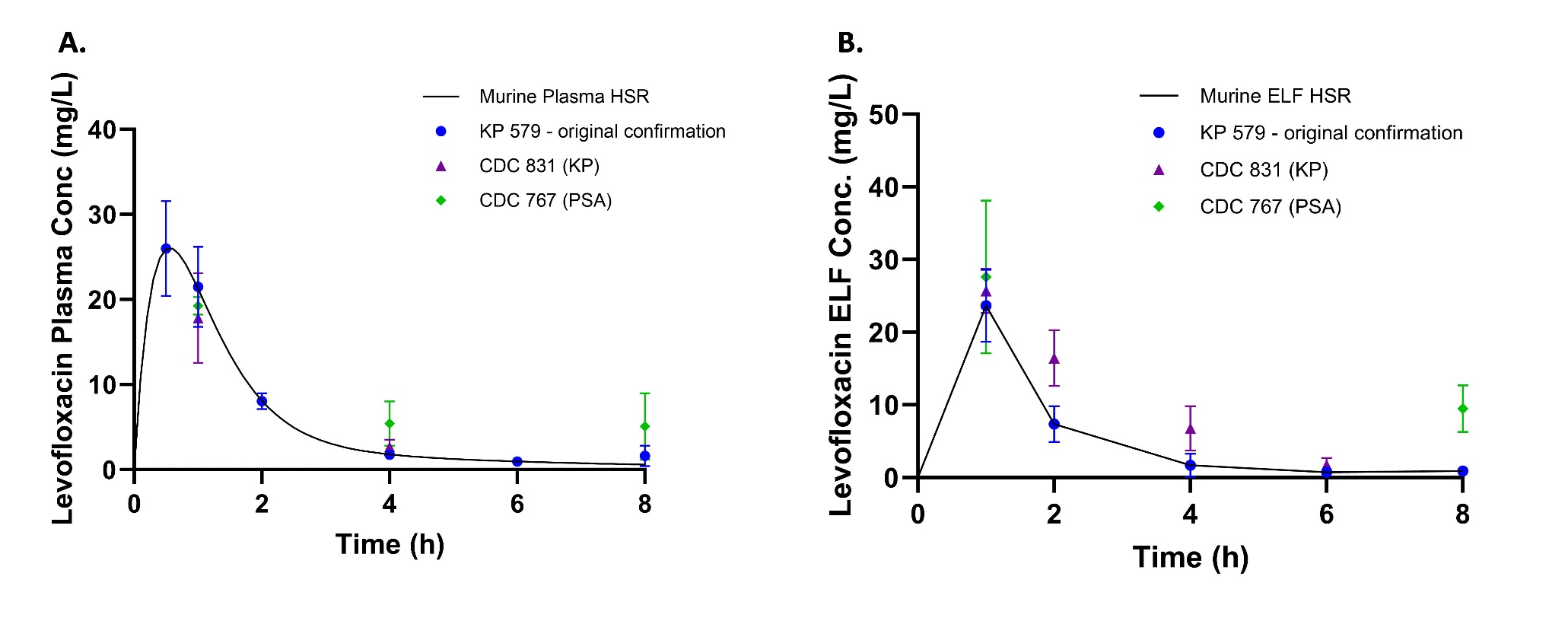

Supplement: dkae333_Supplementary_Data [file dkae333_supplementary_data.docx]
